# Acute Effect of Kinesiology Taping on Postural Stability in Individuals With Unilateral Chronic Ankle Instability

**DOI:** 10.3389/fphys.2020.00192

**Published:** 2020-03-24

**Authors:** Lulu Yin, Lin Wang

**Affiliations:** School of Kinesiology, Shanghai University of Sport, Shanghai, China

**Keywords:** kinesiology taping, chronic ankle instability, postural control, computerized dynamic posturography, perceived stability and comfort

## Abstract

**Background:**

Chronic ankle instability (CAI), which is characterized by deficient postural control, could be improved through kinesiology taping (KT). However, the effect of KT on postural control in CAI individuals is controversial. Therefore, this study aimed to investigate the acute effect of KT on postural control through computerized dynamic posturography (CDP) and self-perceived sensation in CAI individuals.

**Methods:**

Participants with CAI received four different ankle treatments randomly, including KT, athletic taping (AT), sham taping (ST), and no taping (NT). A series of postural stability measurements was performed using CDP subsequently. The measurements included sensory organization test (SOT), unilateral stance (US), limit of stability (LOS), motor control test (MCT), and adaption test (ADT). In addition, self-perceived sensation was measured through visual analog scaling. Repeated measures analysis of variance was conducted to determine whether the difference among KT, AT, ST, and NT was significant; Bonferroni test was used for post hoc analysis.

**Results:**

No significant difference was observed for parameters in SOT, US, and LOS in four different taping treatments. In MCT, the amplitude scaling scores of KT were 35.87% significantly lower than that of NT [p < 0.001, 95% confidence interval (CI) = 0.548–1.795] in forward-small slip and 21.58% significantly lower than that of ST (p = 0.035, 95% CI = 0.089–3.683) in backward-large slip. In ADT, sway energy scores were 7.59% significantly greater in ST than in AT (p = 0.028, 95% CI = −8.343 to −0.320). For perceived stability, KT was significantly greater than ST (p < 0.001, 95% CI = 0.552–1.899) and NT (p < 0.001, 95% CI = 0.797–2.534), and AT was significantly greater than ST (p = 0.001, 95% CI = 0.423–2.246) and NT (p < 0.001, 95% CI = 0.696–2.852). For perceived comfort, KT was significantly greater than AT (p = 0.001, 95% CI = 0.666–3.196) and NT (p = 0.031, 95% CI = 0.074–2.332), and ST was significantly greater than AT (p = 0.007, 95% CI = 0.349–2.931).

**Conclusion:**

KT and AT have limited effect to facilitate postural control for CAI individuals during SOT, US, and LOS. However, KT and AT could provide effective support to cope with sudden perturbation in MCT and ADT. Moreover, KT provided excellent perceived stability and comfort, whereas AT provided excellent perceived stability but least comfort.

## Introduction

Chronic ankle instability (CAI), which is characterized by persistent ankle pain, swelling, feelings of “giving way,” and self-reported disability, has high prevalence during physical exercise (Doherty et al., 2014; Vuurberg et al., 2018). Physical and psychological burden of patients was aggravated by recurrent sprains. CAI develops commonly from lateral ankle sprains, which happened from excessive supination of rearfoot at initial landing (Hertel, 2002), involving typically injury to the lateral ligaments. During the healing process of the ruptured ligaments, the stability of ligaments was destroyed by scar tissues (Jung et al., 2017). Mechanoreceptors in the lateral ligaments are also impaired, obstructing information transmission and resulting in deficient proprioception, peroneal strength (Nanbancha et al., 2019), and motor neuron excitability (Hertel, 2008). Altogether, these changes of neuromuscular control would impair the postural stability of patients with CAI (Hertel, 2008; Cho and Park, 2019).

Postural stability, defined as the ability to control the body center of mass (COM) within a given base of support, is likely the combination of proprioception and neuromuscular control, requiring the integration of somatosensory, visual, and vestibular afferent information (Cathie, 1950; Hertel, 2008). Several meta-analyses (Arnold et al., 2009; Munn et al., 2010) have concluded that postural control in individuals with CAI was weakened. The sway trajectory of the center of pressure (COP) and time to stability increased during single-leg stance tasks (Pope et al., 2011). The decreased efficacy of postural strategies may increase the risk of recurrent sprains and progress to ankle osteoarthritis in CAI patients (Gribble et al., 2016). Currently, high-quality evidence-based therapeutic interventions recommended for CAI including non-steroidal anti-inflammatory drugs, early mobilization, and exercise therapy. In addition, support therapies such as brace and taping are also recommended (Doherty et al., 2017; Kosik et al., 2017).

A recent support intervention that may improve postural control of CAI is kinesiology taping (KT). Owing to its convenient and low-cost feature, KT was used widely in the prevention and treatment of sports injury. Compared with traditional athletic taping (AT), KT is characterized by its elasticity, maintaining the flexibility of joints on the basis of fixation. The elastic property of KT would produce traction and stimulation on the skin and subcutaneous tissues, which may increase sensory input and improve proprioception. Meanwhile, the traction may increase subcutaneous tissue space, thereby improving blood and lymph circulation and alleviating swelling (Cimino et al., 2018; Yang and Lee, 2018). In addition, KT may change muscle alignment, which may promote or inhibit muscle contraction according to the route of muscles (Csapo et al., 2012).

However, current researches that has explored the effect of KT on postural stability of CAI remains controversial. Some researches (Jackson et al., 2016; Andreo et al., 2018; Jelinek et al., 2019) approved that KT could enhance postural stability through reinforcement of ankle stability during functional tasks such as Balance Error Scaling and Y balance test. Conversely, other studies (Shields et al., 2013; de-la-Torre-Domingo et al., 2015) held a negative attitude for effect of KT on postural control. Shields et al. (2013) found no improvement for sway of COP during single stance with an elastic tape. de-la-Torre-Domingo et al. (2015) attributed the improvement of equilibrium to subjective increase in confidence for CAI individuals. On this occasion, it is difficult to draw a definite conclusion for CAI individuals due to large difference in taping duration and method.

Moreover, current assessments on postural control such as single-leg stance and star excursion test evaluate only the effect of task completion but cannot provide objective and comprehensive measurements to describe the trajectory of movements. Computerized dynamic posturography (CDP) evaluates postural stability on the basis of the inverted pendulum model, which records the interrelationship between COP and COM. CDP has been proven to have high reliability and validity and viewed as the “gold standard” for assessing functional postural stability (Harro and Garascia, 2019).

Therefore, our research aimed to investigate the effect of KT on postural stability under sensory organization test (SOT), unilateral stance (US), limit of stability (LOS), motor control test (MCT), adaption test (ADT), and perceived stability and comfort. We hypothesized that taping could improve postural stability and increase perceived stability and comfort of patients with CAI.

## Materials and Methods

### Participants

Considering a power of 0.90, α level of 0.05 in repeated measures analysis of variance (ANOVA), and 0.15 dropout rate, a minimum of 31 participants should be required. A total of 35 male participants (age, 22.97 ± 2.81; height, 1.78 ± 0.06 cm; weight, 73.49 ± 12.33 kg; BMI, 23.27 ± 3.55 kg/m^2^) were recruited from a local university. The inclusion criteria for participants are the following: (1) college students, regular for daily activity (professional athlete or sedentary men were not included); (2) have a history of at least one significant ankle sprain, and the initial sprain occurred at least 12 months before study enrollment; (3) feelings of “giving way” of the injured ankle joint and/or recurrent sprain and/or “feeling of instability”; and (4) a score of Cumberland Ankle Instability Tool questionnaire of <24. Participants who had a history of bilateral sprains, lower limb fracture, operation, nervous and vestibular system disease, or allergic to taping were excluded. All participants were instructed to read and sign an informed consent form. This study was approved by the ethics committee of Shanghai University of Sports.

### Taping Procedure

**FIGURE 1:**
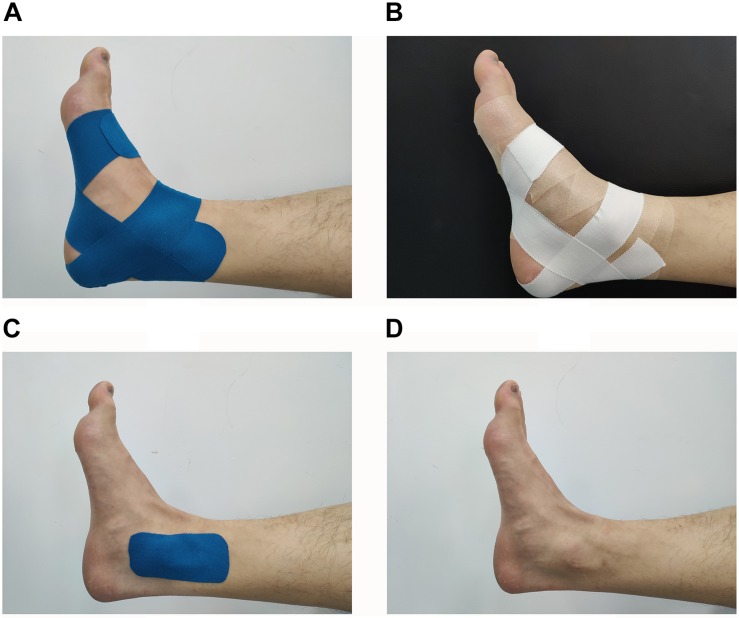
Four different ankle taping treatments. **(A)** KT, kinesiology taping; **(B)** AT, athletic taping; **(C)** ST, sham taping; **(D)** NT, no taping.

Before taping, the taping area should be free of hair and wiped with alcohol. Each participant received four different treatments randomly: real taping, KT and AT; control taping, sham taping (ST); and no taping (NT) (Figure 1).

For KT, we selected the kinesiology tape (50 mm × 5 m) with the ankle balance taping (ABT) method (Kim and Shin, 2017; Lee and Lee, 2017). ABT consists of four strips with 50% tension: (1) posterior talar glide taping, wrapping from the talus to the calcaneus when ankle is in slight dorsiflexion; (2) ankle inversion taping, tape was applied from 5 cm above the medial malleolus, passing by lateral malleolus to the outside of sole when ankle is in slight inversion; (3) ankle eversion taping, tape was applied from 5 cm above the lateral malleolus, passing by medial malleolus to the inside of sole when ankle is in slight eversion; (4) tape was applied to cover 100% of the first strip.

For AT, the athletic tape (50 mm × 13 m) with same method of ABT was used, aiming to find out if the material of the tape would affect performance. For ST, a strip of kinesiology tape (5 cm × 10 cm) without tension was adhered to the medial and lateral malleolus to avoid cutaneous input (Sawkins et al., 2007; Lee and Lee, 2017), aiming to judge the placebo effect of taping. All taping procedures were accomplished by an experienced taping therapist who did not participate in the recruitment and assessment. Moreover, the participants were not told about the function of various taping treatments. The order of taping was counterbalanced and randomized. In addition, 1 week of washout phase was performed between each taping treatment to limit any learning effect. For example, the participant received first random taping treatment on Monday; then, the second random taping treatment would be performed the next Monday, and so on. The total experiment for each participant lasted for 4 weeks.

### Outcome Measures

#### Postural Control

Participants received CDP measurements immediately after taping. CDP measurements were conducted using the NeuroCom Balance Manager System (Version 9.3, Copyright © 1989–2016 Natus Medical Incorporated) SMART EquiTest Mode. Before testing, participants were secured into a harness and stood barefoot on two force plates (23 cm × 46 cm) sampled at 100 Hz, with feet aligned with the platform axis and faced with the visual surround. A screen is embedded on the visual surround to provide feedback to ensure that their center of gravity (COG) was at the center of the target area. The following tests were executed in random sequence.

##### Sensory organization test

This test could differentiate the weight of visual, vestibular, and somatosensory functions in maintaining balance. Participants were required to stand upright as stable as possible to keep their COG steady; each test lasted for 20 s and repeated three times. The six conditions of the SOT test are shown in Table 1. Notably, the “sway referenced” in Table 1 means that the movement of the platform was referenced to the participant’s sway. For example, when a participant leans forward, the platform or visual surround would tilt forward. The theoretical maximum sway without fall in a healthy individual is assumed to be 12.5° (8.25° anterior, 4.25° posterior). The equilibrium score (ES) in each condition was calculated in [12.5 **−** θ(maximum **−** minimum)]/12.5 × 100%, where θ is the maximum anteroposterior COG sway angle recorded in each trial. Moreover, overall composite (COMP) score was the weighted average of all trials and conditions, with greater emphasis given to the more challenging conditions of 4, 5, and 6. Sensory analysis scores [somatosensory (SOM), visual (VIS)] were recorded as well, representing the ability to maintain balance with visual or somatosensory information. All values range from 0 to 100, and values close to 100 indicated greater stability (Harro and Garascia, 2019).

**TABLE 1:** Sensory organization test.

Condition	Eyes	Surface	Surround	Interference	Anticipated response
1	Open	Fixed	Fixed		Somatosensory
2	Closed	Fixed	Fixed	Vision	Somatosensory
3	Open	Fixed	Sway referenced	Vision	Somatosensory
4	Open	Sway referenced	Fixed	Somatosensory	Vision, vestibular
5	Closed	Sway referenced	Fixed	Somatosensory, vision	Vestibular
6	Open	Sway referenced	Sway referenced	Somatosensory, vision	Vestibular

##### Unilateral stance

Participants were required to stand upright by the unstable side leg for 10 s, hands on the anterior superior spine with eyes open or closed. In addition, they were instructed to keep their stance leg extended fully and non-stance leg bent to ∼30° of knee flexion. Three trials were repeated in each condition. The sway velocity of COG (°/s) were exported through the system, and less sway velocities of COG indicated greater instability.

##### Limits of stability

This test quantified the ability to shift their COM to the furthest distance within the base of support. The participants were instructed to stand at the central area represented by a cursor observed on the screen. Once they heard a ring, the participants should initiate their COM to move accurately and quickly into one of the eight target directions (forward, forward-right, right, right-backward, backward, backward-left, left, and left-forward) and maintain their COG to coincide with the target area for 10 s. Target locations were normalized by the subjects’ height. When each direction trial was over, the participants returned to the starting position and await the next trial until all directions were completed. The directional control (DCL) score (%) was calculated as the amount of movement toward the intended direction minus the amount of movement off-axis (Harro and Garascia, 2019).

##### Motor control test

Motor control test evaluates the ability to restore balance for coping with the unexpected anterior–posterior slip of platforms. A sequence of small, medium, and large platform perturbations was delivered in the forward and backward direction. The amplitude of platform slips was scaled to the patient’s height, and no practical trial was given. Direction and amplitude of platform perturbations were randomized, and each trial was repeated for three times. The amplitude scale scores, which is the force exerted on the force plate by the unstable leg in response to the perturbation, were output (Harro and Garascia, 2019).

##### Adaption test

Adaption test analyzed the ability to respond efficiently to unexpected passive dorsiflexion–plantarflexion of ankle. The platform rotated at a velocity of 20°/s in a series of five consecutive rotations in the direction of toes up or down. This test provided a non-dimensional swing energy score (SES), which was determined on the basis of the velocity and acceleration of COP during the first 2 s of perturbation. A smaller SES would represent a greater ability to react more efficiently.

#### Perceived Stability and Comfort

Finally, participants were required to complete visual analog scaling for the comfort and stability of ankle joints during different taping treatments according to their actual feelings during measurements. A score of 0 means “very uncomfortable” and “very unstable,” and 10 means “very comfortable” and “very stable.”

### Statistics

All data were presented as mean ($\bar{x}$) and standard deviation (*s*). Shapiro–Wilk test was used to confirm normal distribution of data. One-way repeated measures ANOVA was conducted to determine whether there was a significant difference among KT, AT, ST, and NT. Bonferroni test was used for *post hoc* analysis. Statistical significance was set at *p* < 0.05. Moreover, 95% confidence interval (CI) was determined, and the effect size was expressed as η^2^. Small effect with 0.01 ≤ η^2^ < 0.06, moderate effect with 0.06 ≤ η^2^ < 0.14, and large effect with η^2^ ≥ 0.14). All statistics were performed with IBM SPSS software (Version 20.0, Chicago, IL, United States).

## Results

The results indicated no significant difference in the four taping treatments for ES under six conditions and COMP in the SOT (Table 2). In the unilateral stance (US) test, the results also showed no significant difference in the four taping treatments for sway velocity of COM (Table 3). Similarly, no significant difference for DCL was found in the four taping treatments in all four directions in the LOS test (Table 3).

**TABLE 2:** Comparison of equilibrium scores and composite scores in sensory organization test (SOT) for different taping treatments ($\bar{x}$ ± s).

		KT	AT	ST	NT	*F* values	*P* values	η^2^
ES	Condition 1	92.70 ± 2.82	92.42 ± 5.8	92.27 ± 5.04	92.64 ± 3.83	0.126	0.898	0.004
	Condition 2	91.45 ± 3.17	90.79 ± 6.15	91.40 ± 2.71	90.50 ± 3.86	0.612	0.516	0.018
	Condition 3	89.79 ± 4.39	91.46 ± 2.70	90.53 ± 3.31	90.0 ± 4.30	2.162	0.116	0.060
	Condition 4	85.32 ± 9.95	86.52 ± 9.43	87.24 ± 8.68	84.63 ± 9.44	0.922	0.418	0.026
	Condition 5	77.65 ± 8.83	78.97 ± 9.55	75.71 ± 9.35	76.25 ± 10.67	1.529	0.212	0.043
	Condition 6	72.04 ± 13.18	76.40 ± 9.69	73.18 ± 11.72	74.38 ± 10.79	1.967	0.139	0.055
COMP		82.86 ± 6.20	84.60 ± 6.20	83.03 ± 6.21	82.80 ± 6.07	1.970	0.123	0.055
VIS		92.03 ± 10.22	93.40 ± 6.82	94.51 ± 7.86	91.34 ± 9.05	1.211	0.310	0.034
SOM		98.71 ± 3.49	98.20 ± 2.39	99.31 ± 5.21	97.77 ± 3.43	1.208	0.308	0.034

*ES, equilibrium scores; COMP, composite scores; VIS, visual scores; SOM, somatosensory scores; A, anterior; P, posterior; L, left; R, right; EO, eyes open; EC, eyes closed.*

**TABLE 3:** Comparison of parameters in US and LOS for different taping treatments ($\bar{x}$ ± *s*).

			KT	AT	ST	NT	*F* values	*P* values	η^2^
US	Sway velocity of COG (°/s)	EO	0.83 ± 0.22	0.89 ± 0.25	0.89 ± 0.29	0.85 ± 0.26	1.225	0.305	0.035
		EC	1.52 ± 0.34	1.70 ± 0.80	1.57 ± 0.51	1.54 ± 0.38	1.265	0.287	0.036
LOS	DCL (%)	A	91.26 ± 4.25	91.17 ± 3.87	91.34 ± 3.98	91.60 ± 4.01	0.106	0.956	0.003
		AR	84.46 ± 7.80	85.60 ± 6.70	85.17 ± 7.85	83.83 ± 9.40	0.441	0.724	0.013
		R	86.66 ± 6.73	88.09 ± 4.87	86.66 ± 5.52	87.43 ± 5.04	0.689	0.533	0.020
		PR	72.71 ± 15.69	73.23 ± 14.24	74.57 ± 13.10	75.40 ± 10.97	0.762	0.518	0.022
		P	82.46 ± 10.49	81.09 ± 9.59	81.54 ± 11.61	82.89 ± 9.38	0.285	0.836	0.008
		PL	72.54 ± 13.70	71.77 ± 12.14	71.57 ± 13.03	73.06 ± 14.0	0.277	0.842	0.008
		L	86.69 ± 6.18	86.83 ± 4.83	86.0 ± 4.69	85.51 ± 7.03	0.518	0.619	0.015
		AL	85.20 ± 7.68	82.94 ± 9.47	85.11 ± 10.65	82.51 ± 10.27	1.572	0.201	0.044

*US, unilateral stance; LOS, limits of stability; DCL, directional control; A, anterior; P, posterior; L, left; R, right; EO, eyes open; EC, eyes closed.*

**FIGURE 2:**
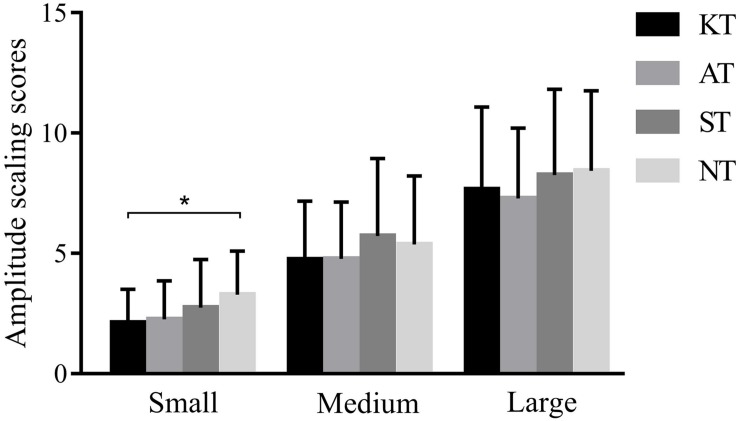
Amplitude scaling scores of four different taping treatments in forward slips in MCT. *p < 0.05.

However, for MCT, we found a significant difference among the four taping treatments in forward-small slip (*p* < 0.001, *F* = 9.304, η^2^ = 0.215). *Post hoc* analysis showed that amplitude scaling scores of KT were 35.87% significantly lower than those of NT (*p* < 0.001, 95% CI = 0.548–1.795) (Figure 2).

**FIGURE 3:**
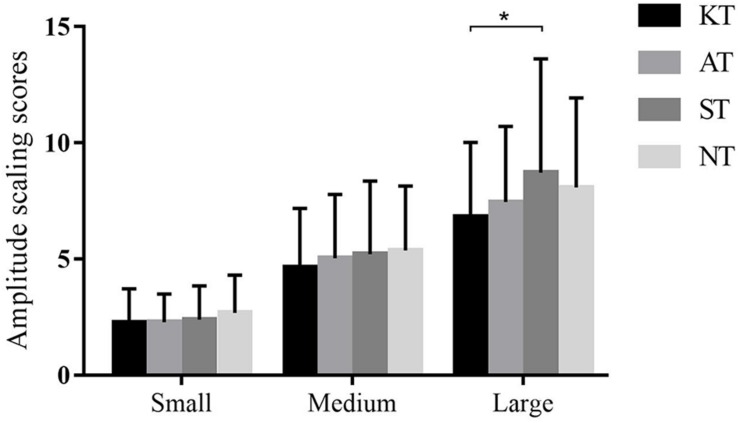
Amplitude scaling scores of four different taping treatments in backward slips in MCT. *p < 0.05.

Similarly, a significant difference was observed among the four taping treatments (*p* = 0.025, *F* = 3.830, η^2^ = 0.101) in backward-large slip. *Post hoc* analysis showed that the amplitude scaling scores of KT were 21.58% significantly lower than those of ST (*p* = 0.035, 95% CI = 0.089–3.683) (Figure 3).

**FIGURE 4:**
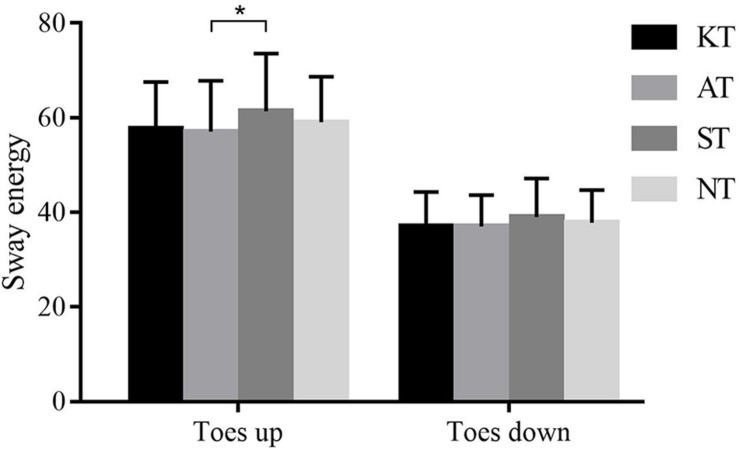
Sway energy of four different taping treatments in ADT. *p < 0.05.

For the SES in toes-up direction measured from ADT, a significant difference was observed among different taping treatments (*p* = 0.044, *F* = 2.799, η^2^ = 0.076). *Post hoc* analysis demonstrated that SES was 7.59% significantly greater in ST than in AT (*p* = 0.028, 95% CI = **−**8.343 to **−**0.320). A similar tendency was observed for SES in toes down although there was no significant difference (Figure 4).

**FIGURE 5:**
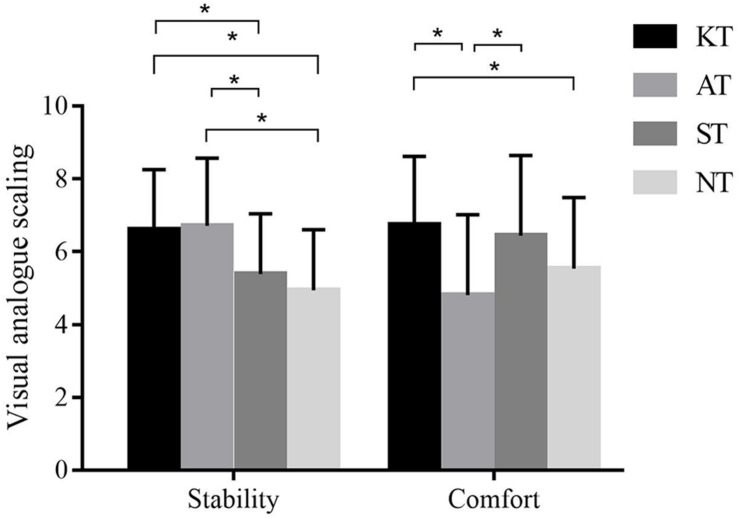
Visual analog scaling of perceived stability and comfort for four different taping treatments. *p < 0.05.

For perceived stability, a significant difference was observed among the different taping treatments (*p* < 0.001, *F* = 16.712, η^2^ = 0.330). *Post hoc* analysis demonstrated that KT was significantly greater than ST (*p* < 0.001, 95% CI = 0.552–1.899) and NT (*p* < 0.001, 95% CI = 0.797–2.534); AT was significantly greater than ST (*p* = 0.001, 95% CI = 0.423–2.246) and NT (*p* < 0.001, 95% CI = 0.696–2.852). For perceived comfort, a significant difference was observed among the different taping treatments (*p* < 0.001, *F* = 9.021, η^2^ = 0.210). *Post hoc* analysis demonstrated that KT was significantly greater than AT (*p* = 0.001, 95% CI = 0.666–3.196) and NT (*p* = 0.031, 95% CI = 0.074–2.332); ST was greater significantly than AT (*p* = 0.007, 95% CI = 0.349–2.931) (Figure 5).

## Discussion

To the best of our knowledge, CDP variables have been verified to have excellent reliability and validity to localize and diagnose pathological mechanisms of deficient postural control (Wrisley et al., 2007; Harro and Garascia, 2019). It provided objective assessment to quantify postural control strategy in the present study. The primary finding of this study was that no significant difference was observed for parameters in SOT, US, and LOS tests. However, KT had significantly lower amplitude scaling scores in MCT, whereas AT has significantly lower sway energy scores in ADT. Furthermore, KT provided significantly greater perceived stability and comfort, whereas AT provided significantly greater stability but smaller perceived comfort.

### Effect of KT on Postural Control With CAI

The results indicated no significant difference for ES in all six conditions, COMP, VIS, and SOM under different taping treatments during SOT, which is inconsistent with our hypothesis. Overall, our results demonstrated that KT and AT could not produce acute effect for double-leg stance for CAI individuals to cope with varied visual, somatosensory, and vestibular environments. Our results were supported by the observation of Lee and Lee (2017), who found no difference among KT, ST, and NT during double-leg stance of Balance Error Scoring System (BESS). However, Jackson et al. (2016) found that KT could improve balance after taping for 48 h compared with the pretaping and control groups. de-la-Torre-Domingo et al. (2015) contributed the improvement of SOT during follow-up to the placebo effect of taping, which increased experience of self-safety.

The interpretation for these conflicting results may lie on the extended effect of taping. Harput and Baltaci (2011) indicated that KT could facilitate muscular strength and jump performance when applied for more than 60 min. Under long-term taping, the CNS may adapt gradually to taping, which could improve postural strategy through sufficient practice (Simon et al., 2014; Jackson et al., 2016). Further research could extend taping duration to find out the optimal duration to improve standing stability for CAI individuals.

Most notably for conditions 2 and 3 where vision was deprived or disturbed, CAI individuals were anticipated to rely more on proprioception for postural stability. Owing to elastic stimulation of KT, it was estimated theoretically to improve proprioception, subsequently exhibiting greater ES than other taping treatments. However, results indicated that KT could not increase ES of conditions 2 and 3. Besides, the sensory analysis scores verified that KT did not change the degree of dependence on visual or proprioceptive system. These findings may be attributed to the mechanism of ABT; the stimulation provided by KT may be insufficient to facilitate deep sensory receptors located in the muscle spindle and tendon organs, just affecting superficial sensation such as tactile and pressure sensation. This assumption was supported by Cimino et al. (2018) who found that the mechanical effect of KT was limited on the superficial skin and Grindstaff et al. (2015) who found that fibular taping did not cause an immediate change in spinal reflex excitability of the soleus and fibularis longus in CAI individuals.

In the case of US test, our results demonstrated no significant difference for sway velocity of COM with both EO and EC under four different taping treatments. This demonstrated that KT and AT could not prompt postural stability during single-leg stance, which was supported by previous studies (Shields et al., 2013; Halim-Kertanegara et al., 2017). Shields et al. (2013) revealed no decisively relevant changes for the COP sway and time to stability between tape conditions. However, Russo et al. (2018) demonstrated that the neuromuscular effect of taping was positive for COP sway through combined exercise and taping in healthy rugby players. The reason for this contradiction was likely that the greater activation of receptors through both exercise and taping provided more afferent input to enhance the stability, compared with taping only in our investigation. Neuromuscular exercise combined with taping in **CAI** should be concentrated in future research.

With regard to limit of stability, DCL scores reflected composite performance of muscle strength, flexibility, and coordination. Surprisingly, no significant difference was observed for DCL scores in all eight directions and COMP score during all different taping treatments. Consistent with our results, Bicici et al. (2012) reported that KT had no significant improvement on star excursion balance test for CAI patients, which confirmed our observation. Delahunt et al. (2010) demonstrated that ankle joint taping was not able to positively influence dynamic postural stability in subjects with CAI. A recent meta-analysis (Tsikopoulos et al., 2019) also demonstrated that taping was not effective in improving dynamic postural control in patients with CAI. Conversely, some research (Andreo et al., 2018; Jelinek et al., 2019) concluded that KT and AT contributed to the balancing action during Y balance test. Difference in measurement methodology may cause contradictory results.

As for MCT, the AS score of KT was 21.58% significantly lower than that of ST in backward-large slip, and the AS score of KT was 35.87% significantly lower than that of NT in forward-small slip. These results indicated that KT did help to maintain postural stability when faced with sudden perturbation. When coping with the unexpected slips, the application of KT could provide flexibility and stability for the ankle, facilitate efficient body response, and thus exhibit smaller sway amplitude of body compared with minimal taping. However, whether this improvement represents clinically meaningful change in the ability to cope with changeable external environments remains unclear.

With regard to ADT, the sway energy scores of AT was lower than that of ST significantly during rotation with toes up. A similar tendency was found during rotation with toes down although not significant. Faced with sudden passive dorsiflexion of tibiotalar joint in ADT, large stability was demanded for the ankle. On this occasion, AT may exhibit superior support than KT. Our result was supported by Briem et al. (2011) who reported that a non-elastic sports tape may enhance dynamic muscle support of the ankle during a sudden inversion perturbation. Our finding revealed that application of taping, regardless of elasticity, could facilitate effective postural response, faced with uneven surface or unexpected alteration of surface in the real environment.

### Effect of KT on Perceived Stability and Comfort With CAI

As for perceived stability and comfort, the results indicated that perceived stability of KT and AT was significantly higher than that of ST and NT, whereas perceived comfort of KT and ST was significantly higher than that of AT. Therefore, KT provided excellent perceived stability and comfort, whereas AT provided excellent perceived stability but least comfort. The finding was in agreement with the results of Long et al. (2017) who reported that KT provided superior comfort than AT, although it was less supportive. Our finding confirmed the psychological effect of KT due to excellent self-experienced stability and comfort, which has been viewed as a potential pathway of working mechanism (Vercelli et al., 2013; Mak et al., 2019). As for AT, the least perceived comfort is likely from poor elasticity property, which may limit the range of motion, affecting acceptance of users.

The consideration for ABT method was based on the instability of the anterior and posterior tibiofibular ligaments CAI individuals. Strips 1 and 4 could limits talus forward displacement and facilitate the improvement of the limited ankle dorsiflexion range of motion. Strip 2 provided reinforcement of lateral ankle ligaments to limit excessive inversion. Strip 3 provided balanced reinforcement for medial ankle to prevent imbalance of ankle. In addition, 50% tension was applied, and it was assumed to facilitate cutaneous receptors of ligaments and provide mechanical stability. However, ABT did not cover peroneal muscle, which may not be able to stimulate the calf muscle group, which should be taken into consideration in future research.

However, limitations must be considered when interpreting the results. First, we did not take the extended effect of taping into consideration; therefore, the interpretation of results in this study were limited to the immediate effect after taping. Future studies should incorporate the extended effect of taping to determine the optimal duration of taping for CAI individuals. Another limitation of our study is that we did not explore postural control difference among healthy individuals, as it is difficult to provide related reference. Future research should conduct comparative analysis of different types of people.

## Conclusion

The present study demonstrated that KT and AT had limited effect to facilitate postural control for CAI individuals during SOT, limit of stability, and unilateral stance. However, KT and AT could provide effective support to cope with perturbation. In addition, KT provided excellent perceived stability and comfort, whereas AT provided excellent perceived stability but the least comfort.

## Data Availability Statement

The datasets generated for this study are available on request to the corresponding author.

## Ethics Statement

The studies involving human participants were reviewed and approved by Ethics Committee of Shanghai University of Sport, iD 2018075. The patients/participants provided their written informed consent to participate in this study.

## Author Contributions

LY recruited the subjects, collected the data, and wrote the manuscript. LW conceived the study, undertook statistical analysis, and interpreted the results.

## Conflict of Interest

The authors declare that the research was conducted in the absence of any commercial or financial relationships that could be construed as a potential conflict of interest.
